# Psychometric Properties of a 17-Item German Language Short Form of the Speech, Spatial, and Qualities of Hearing Scale and Their Correlation to Audiometry in 97 Individuals with Unilateral Menière’s Disease from a Prospective Multicenter Registry

**DOI:** 10.3390/jcm14144953

**Published:** 2025-07-13

**Authors:** Jennifer L. Spiegel, Bernhard Lehnert, Laura Schuller, Irina Adler, Tobias Rader, Tina Brzoska, Bernhard G. Weiss, Martin Canis, Chia-Jung Busch, Friedrich Ihler

**Affiliations:** 1 Department of Otorhinolaryngology, University Hospital, LMU Munich, 81377 Munich, Germany; jennifer.spiegel@med.uni-muenchen.de (J.L.S.); tobias.rader@med.uni-muenchen.de (T.R.); bernhard.weiss@med.uni-muenchen.de (B.G.W.); martin.canis@med.uni-muenchen.de (M.C.); 2 Department of Otolaryngology—Head and Neck Surgery, Sunnybrook Health Sciences Centre, University of Toronto, Toronto, ON M5S 1A1, Canada; 3 Department of Otorhinolaryngology, Head and Neck Surgery, Greifswald University Medicine, 17475 Greifswald, Germany; bernhard.lehnert@med.uni-greifswald.de (B.L.); irina.adler@med.uni-greifswald.de (I.A.); tina.brzoska@med.uni-greifswald.de (T.B.); chia-jung.busch@med.uni-greifswald.de (C.-J.B.)

**Keywords:** patient-reported outcome measures, Menière’s disease, pure tone audiometry, speech audiometry, multicenter prospective patient registry, hearing-specific disability

## Abstract

**Background/Objectives:** Menière’s disease (MD) is a debilitating disorder with episodic and variable ear symptoms. Diagnosis can be challenging, and evidence for therapeutic approaches is low. Furthermore, patients show a unique and fluctuating configuration of audiovestibular impairment. As a psychometric instrument to assess hearing-specific disability is currently lacking, we evaluated a short form of the Speech, Spatial, and Qualities of Hearing Scale (SSQ) in a cohort of patients with MD. **Methods:** Data was collected in the context of a multicenter prospective patient registry intended for the long-term follow up of MD patients. Hearing was assessed by pure tone and speech audiometry. The SSQ was applied in the German language version with 17 items. **Results**: In total, 97 consecutive patients with unilateral MD with a mean age of 56.2 ± 5.0 years were included. A total of 55 individuals (57.3%) were female, and 72 (75.0%) were categorized as having definite MD. The average total score of the SSQ was 6.0 ± 2.1. Cronbach’s alpha for internal consistency was 0.960 for the total score. We did not observe undue floor or ceiling effects. SSQ values showed a statistically negative correlation with hearing thresholds and a statistically positive correlation with speech recognition scores of affected ears. **Conclusions**: The short form of the SSQ provides insight into hearing-specific disability in patients with MD. Therefore, it may be informative regarding disease stage and rehabilitation needs.

## 1. Introduction

Menière’s disease (MD) is characterized by recurrent episodes of vertigo or dizziness, hearing loss, and other aural symptoms. Diagnosing MD can be complicated by the episodic and fluctuating nature of the disease; therefore, the Barany Society has introduced internationally consented diagnostic criteria. By considering the quality and duration of vertigo episodes, audiometry, and a history of other fluctuating ear symptoms, patients are assigned to two categories of diagnostic certainty, namely definite (dMD) or probable MD (pMD) [1]. However, diagnosis remains challenging in many cases, as those criteria may not capture the whole clinical picture of the disease. A considerable share of cases with a subset of characteristic symptoms does not fit into current diagnostic criteria [2]. With a plausible mechanistic explanation for MD pending, potential biomarkers like endolymphatic hydrops, which can be visualized by contrast-enhanced magnetic resonance imaging, still have to prove their clinical value [3,4,5].

A variety of treatment options exist for MD, and the level of evidence is generally low in the field [6,7,8]. Sennaroglou and coworkers were the first to suggest a step-wise approach for cases failing medical treatment. Therefore, intratympanic steroids are preferred due to the lower risk of hearing loss, reserving endolymphatic sac surgery for individuals with good hearing, and considering gentamicin for those with significant hearing loss already [9]. While a preference of conservative and function-preserving options continues, a more comprehensive approach stresses the value of lifestyle modifications, vestibular rehabilitation, and psychotherapy in most if not all cases [10]. In continental Europe, in particular, betahistine is widely prescribed as primary medical treatment. However, it has failed to demonstrate a treatment effect in clinical trials so far [6].

In particular, the recurring bouts of vertigo significantly debilitate patients in their daily life activities, work, and private relationships [11]. The psychological repercussions of vestibular disorders are widely acknowledged [12,13] and can even result in aggravation and prolonging of dizziness [14], suggesting a strong link between psychological factors and quality of life.

Alongside recurrent vertigo episodes, patients experience hearing loss of fluctuating or permanent character. A prerequisite of categorizing patients according to current diagnostic criteria is assessing hearing loss. However, too little attention has been paid to evaluating hearing in MD patients so far [15,16] and, in particular, research focusing on hearing-related quality of life of MD patients is lacking. MD occurs unilaterally in the majority of cases [4,17] and, consecutively, most MD patients develop asymmetric hearing loss, a condition that results in specific impairments in spatial tasks and challenging listening situations [18]. In social settings, patients feel the need to strategically position themselves in a group to optimize their ability to hear and participate [19]. Data suggests that patients with unilateral profound hearing loss suffer a substantial burden of stress associated with increased hearing effort and a range of psychological and social consequences [20]. Common associated struggles include restrictions within social life and sometimes even exclusion, thus resulting in higher levels of handicap [21,22]. As per evaluating advanced hearing situations, the Speech, Spatial, and Quality of Hearing Scale (SSQ) is a commonly applied and validated questionnaire designed to capture the ability and impairment of hearing in daily life [23,24].

In order to gain a deeper understanding of hearing-specific disability in MD patients, available tools are required to be evaluated upon feasibility for this specific cohort. Thus, we aim to explore whether the SSQ is a suitable tool to assess and measure hearing-specific disability in unilateral MD.

## 2. Materials and Methods

### 2.1. Study Design, Sites, and Ethical Considerations

All patients are part of a multicenter prospective patient registry for MD (German title: “Systematische Erfassung von Morbus Menière: Prospektive Beobachtungsstudie”, acronym: “SEMM”). It was initiated in 2021 and is intended for long-term follow up. It is registered with the German Clinical Trial Registry (https://drks.de/) with the ID DRKS00027830. The study was approved by the research ethics boards (REBs) of both study sites (REB approval number Faculty of Medicine of LMU Munich: 21-0779; REB approval number Greifswald University Medicine: BB 055/22), which are both tertiary academic centers with designated academic referral clinics for dizziness. The study was performed according to the current version of the Declaration of Helsinki [25]. Patients were approached for voluntary participation in the patient registry during routine visits to the sites. Inclusion required written informed consent in each case.

### 2.2. Patients and Diagnosis

Patients for the present analysis were included from 2021 to 2024 in Munich and from 2022 to 2023 in Greifswald. All cases received comprehensive vestibular assessment, including clinical examination, videonystagmography, caloric testing, video head impulse test, and posturography. Retrocochlear causes were excluded by MRI of the brain with a focus on the cerebello-pontine angle.

The registry included all patients who fulfilled the criteria for MD according to current diagnostic criteria, which comprise the two diagnostic categories—definite Menière’s disease (dMD) and probable Menière’s disease (pMD) [1]. Exclusion criteria for the registry were vestibular schwannoma in the imaging study, the presence of other vestibular disorders than Menière’s disease, previous intracranial tumor or surgery, stroke, or another central explanation for vestibular symptoms.

Furthermore, all cases that presented with a clinical picture of typical Menière symptoms but did not fit into those canonical categories were included and analyzed as a different diagnostic category (Menière’s Characteristics, MC), as described by Ihler et al. [2]. In brief, this category contains all patients where MD was the most likely explanation after an extensive diagnostic procedure but who did not fulfill the full current diagnostic criteria. In all patients within this category, in the case of any central symptoms, e.g., leading to suspected vestibular migraines, a neurological consultation was performed to rule out central vestibular conditions.

Furthermore, for the analysis at hand, patients with any hint of bilateral Menière’s disease in either anamnesis or diagnostic tests were excluded. Migraine symptoms in those otherwise diagnosed with MD were by itself not an exclusion criterion.

### 2.3. Questionnaire: German SSQ Short Form (SSQ17)

The Speech, Spatial, and Qualities of Hearing Scale (SSQ) questionnaire [23] encompasses the three subscales speech hearing, spatial hearing, and qualities of hearing, originally with a total of 49 questions. The questionnaire is available now in several versions with various numbers of questions and in different languages. Here, the only available German-language version so far was applied in a short form with 17 items [26] to assess patient-reported hearing ability. This version comprises five items for each of the three subscales, as well as two additional questions.

The additional questions are intended to specifically address hearing in quiet environments and listening effort. Additional question 1 asks “You are speaking to another person in a quiet, carpeted room. Can you understand the other person?” (original in German: “*Sie sprechen mit einer anderen Person in einem ruhigen, mit Teppich ausgelegten Raum. Können Sie die andere Person verstehen?*”), while additional question 2 asks “Is it exhausting for you to understand what is being said in a conversation with others?” (original in German: “*Müssen Sie sich sehr anstrengen um zu verstehen, was in einer Unterhaltung mit anderen gesagt wird?*”).

Values for each item were marked on a visual analog scale by the patient. For each item individually, a checkbox was available to mark “not applicable” for the hearing situation in question. Data extraction considered values to one decimal. The values of the SSQ questionnaires were analyzed according to the initial descriptions [23,26]: a total score was calculated as the mean and median of the results of all 17 items. Likewise, mean and median scores for the subscales were obtained. Across all items, higher numeric values signify higher hearing ability with a maximum of 10, while lower values represent increasing impairment with a minimum value of 0.

### 2.4. Pure Tone Audiometry

Audiometric measurements were performed in quiet, soundproof, anechoic chambers by specialized and experienced technical staff. Air-conduction pure tone thresholds were analyzed for each ear separately at 0.25, 0.5, 1.0, 2.0, 3.0, and 4.0 kHz. A summary value was calculated for pure tone audiometry in middle frequencies assumed to be the most relevant for speech understanding. It averaged thresholds for the four frequencies of 0.5, 1.0, 2.0, and 4.0 kHz as assessed by air conduction (4PTA).

### 2.5. Speech Audiometry

Unaided speech recognition of monosyllabic words was assessed at 65 dB with the German-language Freiburg Monosyllabic Test [27,28]. In brief, for each investigation, a list of 20 monosyllabic words recorded from a male speaker was randomly chosen from a total of 20 lists. Monosyllabic words were consecutively presented to the patients for each ear separately via headphones. Each correctly recognized word was accounted for 5%; thus, the maximum recognition score was 100%.

### 2.6. Statistical Analysis

Data was extracted for the intended analysis from the electronic patient records as well as entered and pseudonymized in Microsoft Excel for each study site separately. Merging and clarification were performed by the joint study team until consensus was reached. Data analysis was provided centrally in Greifswald applying R 4.4.1 (https://www.R-project.org/, accessed on 18 January 2024). Pure tone thresholds louder than measurable were set to 120 dB HL. To address missing values in four cases, the following imputations were performed: in one case (1/97, 1.0%), a 4 kHz pure tone air conduction threshold was not available and replaced by the patient’s 3 kHz threshold for the calculation of 4PTA; in three cases (3/97, 3.1%) where speech recognition values were missing, they were imputed via logistic regression from the 4PTA values based on all other ears’ results.

The usual descriptive statistics are reported, using the Gini mean difference (GMD) over standard deviation. Cronbach’s alpha was used to assess the internal consistency of the SSQ questionnaire’s scales. A 95% confidence interval (95% CI) for Cronbach’s alpha was calculated as described before [29] using the implementation in the psych package version 2.4.6 [30]. Locally estimated scatterplot smoothing (LOESS) and Spearman’s correlation coefficients were used to augment scatter plots with nonlinear, non-parametric regression curves [31], using the implementation in ggplot2 [32]. Non-parametric methods were chosen due to the hypothesized monotonic (nonlinear) relationship between variables and potential outliers at distribution extremes. Spearman’s correlation, in particular, robustly quantifies monotonicity, while LOESS visualizes localized trends without assuming global linearity or parametric distributions.

## 3. Results

In total, 97 consecutive patients were included in the analysis. In 59 cases (60.8%), a majority was from the larger study center. The largest share of patients (72/97, 74.2%) accounted for definite MD (dMD). The mean age of all patients was 56.2 ± 15.0 years. Age and affected side were distributed evenly between diagnostic categories. Within the total cohort, the majority of cases were female (56.7%), with a borderline significant difference between diagnostic categories. The majority of patients (50/97, 51.6%) were users of hearing aids. Hearing aid use was significantly more prevalent in the diagnostic category dMD (Table 1).

**Figure 1:**
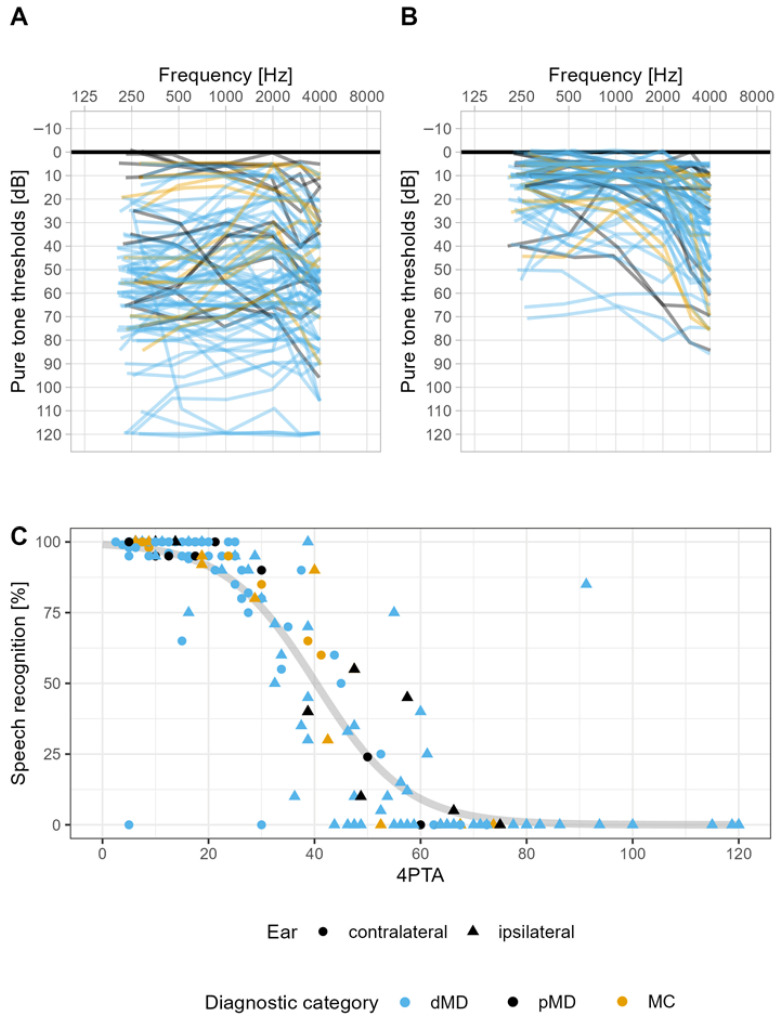
Overview of pure tone and speech audiometry results for the total cohort (*n* = 97). Each individual’s pure tone air-conduction thresholds [dB HL] on affected (**A**) and non-affected (**B**) ears. Analysis (logistic regression) of a correlation between frequency pure tone threshold average (4PTA) and speech recognition scores (Freiburg Monosyllabic Test) (**C**).

Hearing loss as assessed was asymmetric in most cases. Pure tone thresholds were worse in the affected ears, with a mean 4PTA of 51.9 ± 30.2 dB HL (Figure 1A) compared to 19.3 ± 15.0 dB HL in the non-affected ears (Figure 1B). The mean difference between affected and unaffected ears was 32.6 ± 25.2 dB. A total of 70 cases (72.2%) had an interaural difference of more than 15 dB and 51 (52.6%) had a difference of more than 30 dB. Speech audiometry revealed severely impaired speech recognition in affected ears, with an average recognition score for monosyllables of 33.9% ± 42.9%. In contrast, unaffected ears achieved 86.6% ± 21.6%. There was a strong sigmoid correlation between 4PTA and the speech recognition score (*p* < 0.01) in the logistic regression model (Figure 1C). A breakdown of audiometric data by diagnostic categories revealed that patients from dMD showed significantly worse hearing when assessed by pure tone, as well as speech audiometry compared to the other diagnostic categories (Supplementary Table S1).

**Figure 2:**
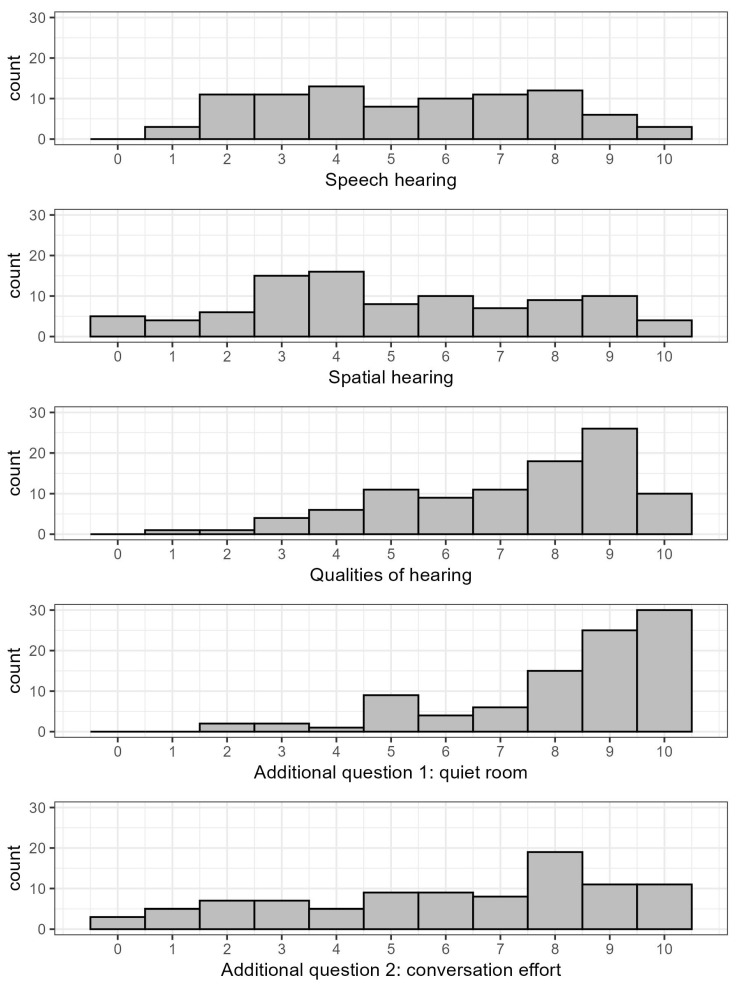
Distribution of SSQ results within subscales (average of responses for the respective five items) and two single-item additional questions. Absolute count of values per interval in the total investigated cohort (*n* = 97).

In the whole investigated cohort, the mean total score of the SSQ17 was 6.0 ± 2.1 (median 5.9, range 1.6–10.0, skew 0.0). The mean score of the subscale speech hearing was 5.3 ± 2.5 (median 5.4, range 0.5–10.0, skew 0.1), spatial hearing was 5.1 ± 2.7 (median 4.6, range 0.0–10.0, skew 0.1), and qualities of hearing was 7.2 ± 2.1 (median 7.8, range 1.2–10.0, skew −0.7). Additional questions 1 and 2 yielded a mean of 8.2 ± 2.0 (median 9.0, range 1.8–10.0, skew −1.3) and 6.1 ± 2.9 (median 7.0, range 0.0–10.0, skew −0.5), respectively. For the subscales, as well as additional question 2, we did not notice floor or ceiling effects. Skew was, however, markedly to the left for both the subscale qualities of hearing, as well as additional question 2, indicating a predominance of individuals with high scores in the respective task. Additional question 1 showed the highest skew to the left and an additional ceiling effect of the values. This signified that most individuals indicated good performance in the comparably simple listening situation with one conversational partner in a quiet room without reverberation (Figure 2). Analysis of SSQ17 results by diagnostic category showed no statistically significant differences between the respective subcohorts, neither for the total score nor for domains or additional questions (Table 2).

To assess the applicability of the SSQ17 for this particular patient cohort with MD, we calculated Cronbach’s alpha for the total score and for the subscales. For the total score, Cronbach’s alpha was 0.960 (95% CI 0.95; 0.97). Speech hearing showed 0.936 (95% CI 0.91; 0.95), spatial hearing 0.956 (95% CI 0.94; 0.97), and quality of hearing 0.904 (95% CI 0.87; 0.93). Therefore, Cronbach’s alpha showed excellent values for the total score and all three subscales, affirming the value of each individual item and validating the applicability for patients with Menière’s disease. A breakdown of the consistency analysis to subscales and single items is given in Supplementary Table S2. We also assessed correlations between subscales and additional questions. Statistically significant strong positive correlations were found between speech hearing and spatial hearing with *R* = 0.755, quality of hearing with *shou* = 0.706, and additional question 2 with *R* = 0.705. All other pairwise correlations were positive as well but less pronounced.

**Figure 3:**
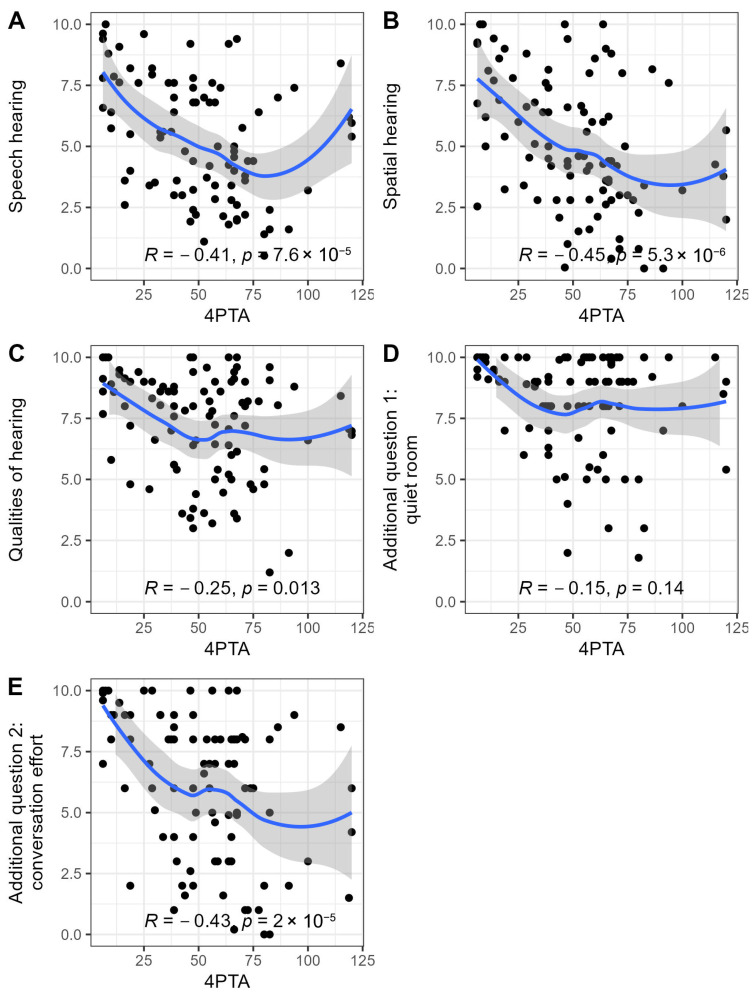
Correlations between SSQ subscales/additional questions and mid-frequency pure-tone threshold average (4PTA). (**A**–**C**) Subscales; (**D**,**E**) additional questions; blue line: LOESS smoother; *R*: Spearman correlation coefficient.

Finally, we explored a correlation between audiometric variables, as well as SSQ17 subscales and additional questions. We supposed higher thresholds in pure tone audiometry might transmit to lower SSQ values, as both aim to quantify hearing impairment. Therefore, we calculated correlations between SSQ17 responses and 4PTA on affected ears, excluding two outliers with very large 4PTA results. Statistically highly significant moderate negative correlations were seen for speech hearing with *R* = −0.41 (*p* < 0.001), for spatial hearing with *R* = −0.45 (*p* < 0.001), and for additional question 2 with *R* = −0.43 (*p* < 0.001). Furthermore, quality of hearing (*R* = −0.25, *p* = 0.013) showed a weak negative correlation (Figure 3).

**Figure 4:**
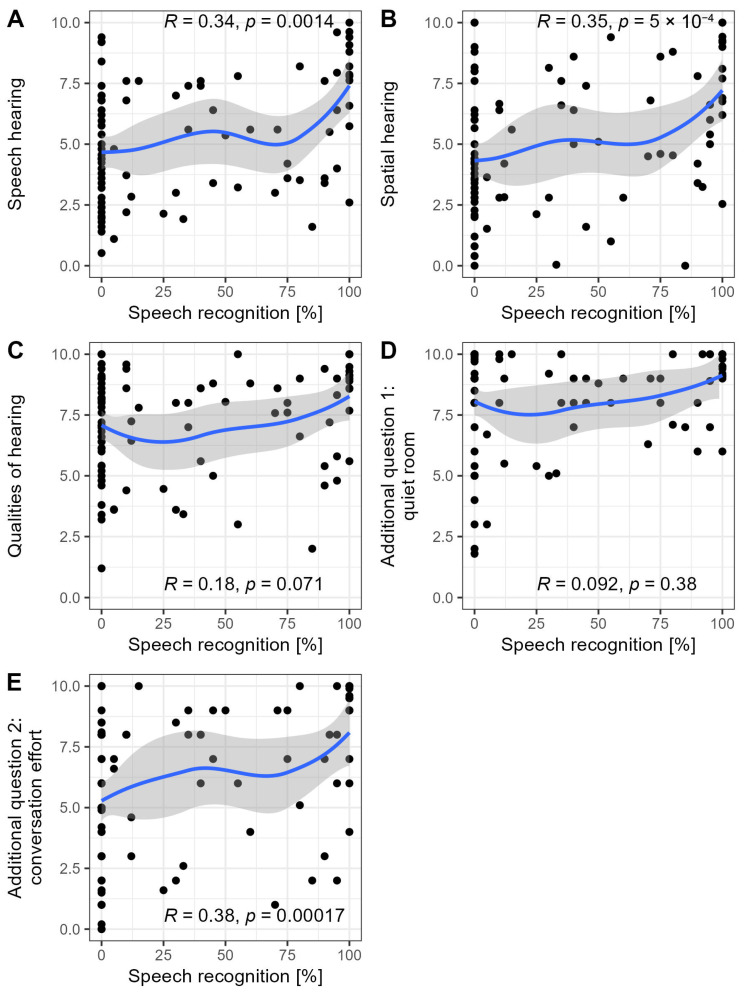
Correlations between SSQ subscales/additional questions and speech recognition scores (Freiburg Monosyllabic Test). (**A**–**C**) Subscales; (**D**,**E**) additional questions; blue line: LOESS smoother; R: Spearman correlation coefficient.

Speech audiometry was considered, as it may be more responsive to impaired communication skills due to hearing loss. However, correlations of speech recognition scores and SSQ are visually less clear due to the distribution being skewed with an accumulation of observations at 0% speech recognition. We noted statistically significant-to-highly significant moderate positive relationships for the subscales in speech hearing (R = 0.34, *p* < 0.01) and spatial hearing (R = 0.35, *p* < 0.001), as well as for additional question 2 (R = 0.38, *p* < 0.001), with the speech recognition scores of affected ears (Figure 4).

## 4. Discussion

This study represents the first analysis of the Speech, Spatial, and Qualities of Hearing Scale (SSQ) in a large cohort of patients with MD. We demonstrate that the SSQ17 is a viable and reliable tool for assessing hearing-related disability in this population, exhibiting excellent internal consistency (Cronbach’s alpha > 0.90). SSQ subscales and additional questions negatively correlated with pure tone thresholds and positively correlated with speech recognition scores, highlighting its ability to capture aspects representing both tonal thresholds and more complex speech recognition.

So far, the analysis of SSQ results in MD patients has been only performed in the follow-up after cochlear implantation [33,34,35]. The consideration of a more representative population for that condition is therefore this study’s unique earmark. Current standard audiological assessments for MD primarily rely on pure tone and speech audiometry. While valuable, these measures do not fully capture the patient’s perceived disability. Our findings underscore the value of incorporating a patient-reported outcome measure (PROM) like the SSQ to obtain a more comprehensive assessment of the detrimental effects of MD on daily life and advanced auditory functions. The demographic characteristics of our cohort (mean age 56.2 ± 15.0 years, 57% female) align with those reported in other large MD cohorts [36,37].

The SSQ is an established questionnaire alongside the Abbreviated Profile of Hearing Aid Benefit (APHAB) for evaluating the impact of hearing loss and the effect of hearing rehabilitation on quality of life [38]. Its sensitivity in advanced listening conditions, such as speech perception in noise and sound localization, is particularly noteworthy [39]. With Cronbach’s alpha of 0.960 for the total score and values from 0.904 to 0.956 in the subscales, we see the SSQ as a reliable instrument both in total and with regard to the subscales and their implied latent variables. For the English version, a threshold of 0.9 has been suggested for the clinical applicability for the instrument in whole, as well as for the three subscales individually [40]. Regarding clinical relevance, our results align with the principle that a minimum of two standard deviationd difference from a healthy population is a reasonable threshold for defining hearing-related disability Therefore, thresholds of 7.25 for the total score, as well as 6.84 for the speech hearing, 6.14 for the spatial hearing, and 8.18 for the quality of hearing subscale, have been reported [41]. The observed SSQ scores in our MD cohort are clearly below this threshold, indicative of pathological hearing impairment.

It is likely that differences between study populations strongly affect the results obtained by a patient-reported outcome measure. Regarding age, higher SSQ scores have been reported from younger populations previously [42]. However, our MD cohort demonstrated significantly worse scores, even when compared to a normative sample of similar age [43], indicating a substantial additional impact of the disease on perceived hearing ability.

Already early after development, the SSQ demonstrated its utility in assessing asymmetric hearing loss. Interestingly, however, our results with asymmetric hearing loss due to MD are slightly above an SSQ total score of 5.9 given for 103 individuals with symmetric and markedly above 4.7 for 50 individuals with asymmetric hearing loss, assessed in an unaided condition and without specified etiology in both groups [24]. Therefore, not only asymmetry but also the underlying condition has to be considered. Hearing asymmetry might also explain the specific profile of our results with marked skew in the subscale qualities of hearing, as well as in both additional questions.

Many studies assessed the benefit of an intervention, like the provision of hearing aids, by the SSQ. A comparably recent study investigated two groups with 45 and 41 adults aged 60 years and older with symmetric bilateral moderate hearing loss. Applying a Danish-language SSQ version with 12 items, they found an unaided baseline value of 4.2 and 3.9, 6.3 and 6.7, and 5.6 and 5.9 for the speech, spatial, and qualities of hearing scales, respectively. Two months after fitting basic hearing aids, those values increased to 5.6, 7.4, and 6.9. Compared to that, premium hearing aids were able to achieve an additional benefit, resulting in values of 7.0, 7.8, and 7.7, representing a significant difference in the speech and qualities of hearing scales [44]. Adapting those values for comparison to the present study, results for the subscales were in keeping with unaided hearing-impaired individuals. That being said, however, our cohort represents a mix regarding hearing rehabilitation, with a slight majority (50/97, 51.6%) of current hearing aid users. Taken together, population characteristics regarding age as well as etiology, type of hearing loss, and rehabilitation status should be considered when the SSQ results and profile are compared between different studies. Future investigations regarding asymmetric hearing loss should, in particular, consider causes for our interestingly disparate results with the additional questions.

Although MD significantly affects hearing, the debilitating effects of vertigo often overshadow these auditory symptoms from the patient perspective. This has been described by an assessment of illness intrusiveness in 74 cases with MD, where vertigo had the greatest detrimental effect on daily life, followed by hearing loss and tinnitus [36]. The dizziness-specific quality of life instrument Dizziness Handicap Inventory illustrates a mild handicap in MD patients, with a statistically significant worse value for bilateral cases [36,45]. With the Vertigo Symptom Scale, Yardley and coworkers described vertigo severity as the most prominent factor for impaired generic quality of life in a community-based sample of individuals with MD [11].

Future research should consider incorporating multimodal assessments—probably by combining instruments for both auditory and vestibular symptoms—to capture the holistic impact of MD on patient well-being. Validated disease-specific instruments, such as the Menière’s Disease Outcome Questionnaire and the Menière’s Disease Quality of Life (MenQoL) questionnaire, could provide valuable complementary information [46,47], but these are not yet available in a validated German-language version. The “Ménière’s Disease Patient-Oriented Symptom-Severity Index” [36,48] has recently been validated in German [49] and might be a promising option in this regard in the future. However, SSQ remains a validated instrument for the assessment of hearing disability, broadly applicable to populations with a variety of etiologies, and it can be used alongside these more disease-specific instruments.

This study’s findings highlight the importance of considering the unique characteristics of MD, such as fluctuating symptoms and asymmetric hearing loss, when interpreting PROMs. Longitudinal studies are needed to further investigate the changes in SSQ scores over time and to determine the factors that contribute to variability in patient-reported outcomes. In conclusion, this study demonstrates that the SSQ is a valuable and reliable instrument for assessing hearing-related disability in MD patients. Incorporating PROMs like the SSQ into routine clinical practice can facilitate a more comprehensive understanding of the impact of MD on patients’ communication abilities and inform individualized treatment plans. Further research should focus on integrating audiometric and vestibular assessments with PROMs to characterize the broad spectrum of MD symptoms and disabilities in even more detail.

## Figures and Tables

**Table 1:** Patient’s characteristics by diagnostic category.

Characteristic	dMD, *n* = 72 (74%) ^1^	pMD, *n* = 14 (14%) ^1^	MC, *n* = 11 (11%) ^1^	*p*-Value ^2^
Study center				0.3
Greifswald	29 (40%)	7 (50%)	2 (18%)	
Munich	43 (60%)	7 (50%)	9 (82%)	
Affected side				0.5
Left	42 (58%)	6 (43%)	5 (45%)	
Right	30 (42%)	8 (57%)	6 (55%)	
Sex				0.050
M	34 (47%)	2 (14%)	6 (55%)	
F	38 (53%)	12 (86%)	5 (45%)	
Age [yrs]	57 (50, 66)	57 (54, 70)	56 (43, 60)	0.6
Hearing aid user	43 (61%)	3 (21%)	4 (36%)	0.012
Missing	2	0	0	

^1^ *n* (%), median (interquartile range); ^2^ Fisher’s exact test; Kruskal–Wallis rank sum test; Pearson’s chi-squared test; canonical diagnostic categories: dMD, definite Menière’s disease; pMD, probable Menière’s disease [1]; experimental category MC, Menière’s characteristics [2].

**Table 2:** Results of the SSQ by diagnostic category.

	dMD, *n* = 72 ^1^	pMD, *n* = 14 ^1^	MC, *n* = 11 ^1^	*p*-Value ^2^
total score	5.9 (4.2, 7.5)	7.2 (4.1, 9.4)	5.8 (4.5, 7.8)	0.7
missing	8	3	2	
speech hearing subscale	5.0 (3.0, 7.0)	6.2 (3.9, 8.4)	6.3 (3.6, 7.8)	0.3
missing	6	2	1	
spatial hearing subscale	4.5 (2.8, 6.9)	4.3 (2.8, 7.7)	5.0 (4.2, 8.8)	0.5
missing	3	0	0	
qualities of hearing subscale	8.0 (6.3, 8.9)	7.0 (4.6, 9.1)	7.2 (4.8, 9.0)	0.8
missing	0	0	0	
additional question 1: quiet room	9.0 (8.0, 10.0)	9.0 (8.0, 9.5)	8.0 (7.0, 10.0)	0.9
missing	1	1	1	
additional question 2: conversational effort	6.0 (3.0, 8.1)	8.0 (7.0, 9.5)	6.5 (3.0, 8.0)	0.057
missing	1	1	1	

^1^ *n* (%), median (interquartile range); ^2^ Fisher’s exact test; Kruskal–Wallis rank sum test; Pearson’s chi-squared test; canonical diagnostic categories: dMD, definite Menière’s disease; pMD, probable Menière’s disease [1]; experimental category MC, Menière’s characteristics [2].

## Data Availability

The data that supports the findings of this study cannot be made openly available due to national data protection laws. However, upon individual reasonable request and for scientific purposes only, the author team will provide an anonymized dataset.

## Supplementary Materials

The following supporting information can be downloaded at: https://www.mdpi.com/article/10.3390/jcm14144953/s1, Supplementary Table S1: Audiometry by diagnostic category. Supplementary Table S2: Internal consistency of the multi-item subscales of the SSQ.
