# Supplementary material for: Psychometric Properties of a 17-Item German Language Short Form of the Speech, Spatial, and Qualities of Hearing Scale and Their Correlation to Audiometry in 97 Individuals with Unilateral Menière’s Disease from a Prospective Multicenter Registry
Source: J Clin Med. 2025 Jul 13;14(14):4953. doi: 10.3390/jcm14144953 (PMC12294874; doi:10.3390/jcm14144953)
Supplement: Supplementary file 1 [file jcm-14-04953-s001.zip › 250712 Supplementary Table S1.pdf]

"Psychometric Properties of a 17-Item German Language Short Form of the Speech, Spatial, and Qualities of Hearing Scale and Their Correlation to Audiometry in 97 Individuals with Unilateral Menière's Disease from a Prospective Multicenter Registry"

by Jennifer L. Spiegel, Bernhard Lehnert, Laura Schuller, Irina Adler, Tobias Rader, Tina Brzoska, Bernhard G. Weiss, Martin Canis, Chia-Jung Busch, Friedrich Ihler

Supplementary Table 1: Audiometry by diagnostic categories

**Test and side**	**dMD**, *n* = 72[1]	**pMD**, *n* = 14[1]	**MC**, *n* = 11[1]	***p*-value**[2]
4PTA affected ear [dB HL]	58 (39, 71)	39 (10, 58)	40 (19, 53)	0.005
4PTA unaffected ear [dB HL]	16 (9, 25)	11 (8, 21)	13 (8, 30)	0.7
speech recognition score affected ear [%]	0 (0, 48)	50 (10, 100)	80 (0, 95)	0.002
speech recognition score unaffected ear [%]	98 (90, 100)	100 (95, 100)	100 (85, 100)	0.8

[1]Median (interquartile range);

[2]Fisher's exact test; Kruskal-Wallis rank sum test; Pearson's Chi-squared test;

Canonical diagnostic categories dMD, definite Menière's disease; pMD, probable Menière's disease [1]; experimental category MC, Menière's Characteristics [2].
