# Supplementary material for: Psychometric Properties of a 17-Item German Language Short Form of the Speech, Spatial, and Qualities of Hearing Scale and Their Correlation to Audiometry in 97 Individuals with Unilateral Menière’s Disease from a Prospective Multicenter Registry
Source: J Clin Med. 2025 Jul 13;14(14):4953. doi: 10.3390/jcm14144953 (PMC12294874; doi:10.3390/jcm14144953)
Supplement: Supplementary file 1 [file jcm-14-04953-s001.zip › 250712 Supplementary Table S2.pdf]

"Psychometric Properties of a 17-Item German Language Short Form of the Speech, Spatial, and Qualities of Hearing Scale and Their Correlation to Audiometry in 97 Individuals with Unilateral Menière's Disease from a Prospective Multicenter Registry"

by Jennifer L. Spiegel, Bernhard Lehnert, Laura Schuller, Irina Adler, Tobias Rader, Tina Brzoska, Bernhard G. Weiss, Martin Canis, Chia-Jung Busch, Friedrich Ihler

Supplementary Table 2: Internal consistency of the multi-item subscales of the SSQ17

Subscale	Item #	Missing	Mean	SD	Skew	Item Difficulty	Item Discrimination	Cronbach's alpha subscale if item deleted	Mean inter-item correlation	Cronbach's alpha subscale
Speech hearing	1	0.0 %	5.2	2.7	0.1	0.5	0.82	0.92	0.748	0.936
	2	0.0 %	5.6	2.6	-0.1	0.6	0.83	0.92		
	3	4.1 %	5.3	2.8	0.0	0.5	0.88	0.91		
	4	4.1 %	5.8	2.5	-0.1	0.6	0.81	0.93		
	5	1.0 %	4.8	2.9	0.1	0.5	0.82	0.92		
Spatial hearing	1	0.0 %	4.7	3.0	0.2	0.5	0.88	0.94	0.812	0.956
	2	1.0 %	5.2	3.0	-0.1	0.5	0.87	0.95		
	3	1.0 %	5.1	3.0	-0.1	0.5	0.90	0.94		
	4	1.0 %	4.9	2.8	0.3	0.5	0.88	0.94		
	5	3.1 %	5.4	2.8	-0.0	0.54	0.85	0.95		
Qualities of hearing	1	0.0 %	6.7	2.5	-0.6	0.67	0.73	0.89	0.652	0.904
	2	0.0 %	7.4	2.3	-0.7	0.74	0.73	0.89		
	3	0.0 %	7.1	2.5	-0.9	0.71	0.77	0.88		
	4	0.0 %	7.4	2.5	-1.1	0.74	0.78	0.88		
	5	0.0 %	7.5	2.5	-1.1	0.75	0.78	0.88		

SD, standard deviation.
